# (η^6^-Benzene)­dichlorido­(chloro­dicyclo­hexyl­phosphane-κ*P*)ruthenium(II) chloro­form monosolvate

**DOI:** 10.1107/S1600536814012975

**Published:** 2014-06-11

**Authors:** Saravanan Gowrisankar, Helfried Neumann, Anke Spannenberg, Matthias Beller

**Affiliations:** a Division of Organic Chemistry, Institute of Chemical and Engineering Sciences, 8 Biomedical Grove, Neuros, #07-01, 138665, Singapore; b Leibniz-Institut für Katalyse e. V. an der Universität Rostock, Albert-Einstein-Strasse 29a, 18059 Rostock, Germany

## Abstract

The title compound, [RuCl_2_(η^6^-C_6_H_6_)(C_12_H_22_ClP)]·CHCl_3_, was prepared by reaction of [RuCl_2_(η^6^-C_6_H_6_)]_2_ with chloro­dicyclo­hexyl­phosphane in CHCl_3_ at 323 K under argon. The Ru^II^ atom is surrounded by one arene ligand, two Cl atoms and a phosphane ligand in a piano-stool geometry. The phosphane ligand is linked by the P atom, with an Ru—P bond length of 2.3247 (4) Å. Both cyclo­hexyl rings at the P atom adopt a chair conformation. In the crystal, the Ru^II^ complex mol­ecule and the chloro­form solvent mol­ecule are linked by a bifurcated C—H⋯(Cl,Cl) hydrogen bond. Intra­molecular C—H⋯Cl hydrogen bonds are also observed.

## Related literature   

For the mol­ecular structure of Ru complexes with the related chloro­diphenyl­phosphane ligand, see: Jantscher *et al.* (2009 ▶); Torres-Lubián *et al.* (1999 ▶).
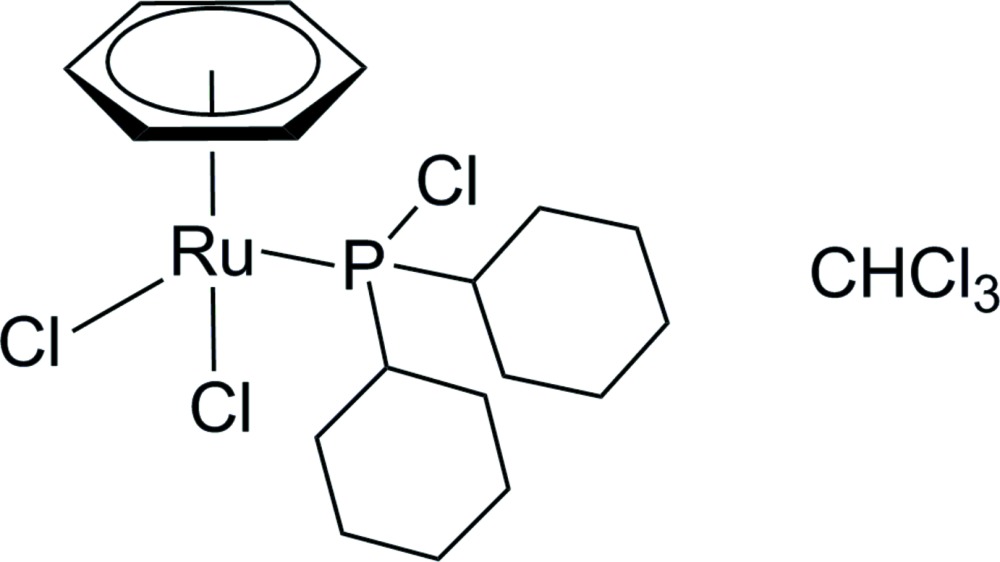


## Experimental   

### 

#### Crystal data  

[RuCl_2_(C_6_H_6_)(C_12_H_22_ClP)]·CHCl_3_
*M* *_r_* = 602.16
Monoclinic, 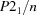

*a* = 7.9717 (1) Å
*b* = 16.3020 (2) Å
*c* = 18.0602 (3) Å
β = 91.244 (1)°
*V* = 2346.45 (6) Å^3^
*Z* = 4
Mo *K*α radiation
μ = 1.42 mm^−1^
*T* = 150 K
0.36 × 0.22 × 0.11 mm

#### Data collection  

Bruker Kappa APEXII DUO diffractometer
Absorption correction: multi-scan (*SADABS*; Bruker, 2008 ▶) *T*_min_ = 0.630, *T*_max_ = 0.859
37052 measured reflections
5619 independent reflections
4963 reflections with *I* > 2σ(*I*)
*R*_int_ = 0.031

#### Refinement  

*R*[*F* ^2^ > 2σ(*F* ^2^)] = 0.019
*wR*(*F* ^2^) = 0.048
*S* = 1.05
5619 reflections
244 parameters
H-atom parameters constrained
Δρ_max_ = 0.41 e Å^−3^
Δρ_min_ = −0.37 e Å^−3^

### 

Data collection: *APEX2* (Bruker, 2011 ▶); cell refinement: *SAINT* (Bruker, 2009 ▶); data reduction: *SAINT*; program(s) used to solve structure: *SHELXS97* (Sheldrick, 2008 ▶); program(s) used to refine structure: *SHELXL97* (Sheldrick, 2008 ▶); molecular graphics: *XP* in *SHELXTL* (Sheldrick, 2008 ▶); software used to prepare material for publication: *SHELXL97*.

## Supplementary Material

Crystal structure: contains datablock(s) I, Global. DOI: 10.1107/S1600536814012975/is5363sup1.cif

Structure factors: contains datablock(s) I. DOI: 10.1107/S1600536814012975/is5363Isup2.hkl

CCDC reference: 1006720

Additional supporting information:  crystallographic information; 3D view; checkCIF report

## Figures and Tables

**Table 1:** Hydrogen-bond geometry (Å, °)

*D*—H⋯*A*	*D*—H	H⋯*A*	*D*⋯*A*	*D*—H⋯*A*
C14—H14*B*⋯Cl1	0.99	2.56	3.4080 (17)	144
C18—H18*A*⋯Cl1	0.99	2.74	3.5366 (17)	138
C19—H19⋯Cl1^i^	1.00	2.69	3.5539 (17)	144
C19—H19⋯Cl2^i^	1.00	2.77	3.6119 (18)	142

Symmetry code: (i) 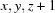.

**Fig. 1.**
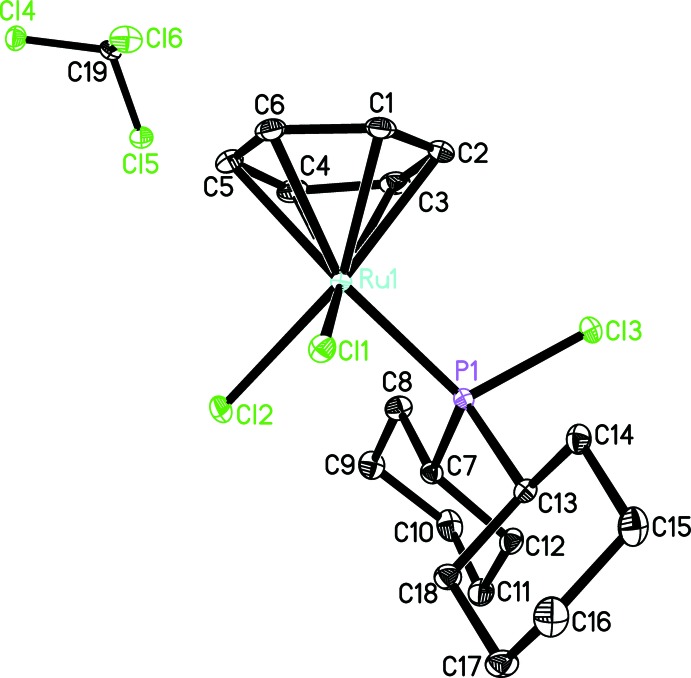
The molecular structure of the title compound with 30% displacement ellipsoids. Hydrogen atoms are omitted for clarity.

## supplementary crystallographic information

### S1. Comment

The half-sandwich (η^6^–C_6_H_6_)-dichlorido(chlorodicyclohexylphosphane)ruthenium(II) complex was formed by reaction of one equivalent of [RuCl_2_(η^6^-C_6_H_6_)]_2_ with two equivalents of (Cy_2_P(1-naphthoyl)) ligand under hydrogenation conditions (CHCl_3_, 60 bar of H_2_, 353 K, 3 hrs) as a side product. The cleavage of the 1-naphthoyl group from [RuCl_2_(η^6^-C_6_H_6_)(Cy_2_P(1-naphthoyl)] complex forms firstly [RuCl_2_(η^6^-C_6_H_6_)(Cy_2_PH)] and subsequent chlorination of the dicyclohexylphosphane unit due to CHCl_3_ yields the title compound in poor yield. Additionally, we could not observe any trace amount of title compound by using non-chlorinated solvents such as MeOH. The substitution of hydrogen next to phosphane by chlorine coming from solvent molecules is also described for the formation of a Ru-complex with the related chlorodiphenylphosphane ligand by Torres-Lubián *et al.* (1999). More specifically, the title complex was formed by reaction of [RuCl_2_(η^6^-C_6_H_6_)]_2_ with chlorodicyclohexylphosphane in CHCl_3_ at 323 K under argon in 41% yield. Crystals suitable for X-ray crystal structure analysis could be obtained by crystallization from a chloroform/heptane mixture. In the ^31^P NMR spectrum of the complex the signal for the phosphorus was observed at 156.3 p.p.m., whereas free ligand signal appears at 128.8 p.p.m.. The title compound shows the three legged piano-stool geometry at the ruthenium centre with the arene, chlorodicyclohexylphosphane and two chlorine ligands in the coordination sphere (Fig. 1). The phosphane ligand is linked by the phosphorus with a Ru—P bond length of 2.3247 (4) Å. Both cyclohexyl rings at the phosphorus atom adopt a chair conformation. The Ru complex is co-crystallized with CHCl_3_.

### S2. Experimental

A 50 ml round bottom flask with inert gas valve was charged with 0.05 mmol (25 mg) [RuCl_2_(η^6^-C_6_H_6_)]_2_ and 4 ml CHCl_3_ under argon atmosphere. To this suspension 0.105 mmol (21 µ*L*) chlorodicyclohexylphosphane was added and the reaction mixture was allowed to react 3 h at 323 K. A clear red brown solution has been formed and the volume was reduced carefully in high vacuum to *ca* 1 ml. Next 20 ml heptane was added to the reaction mixture and cooled for 1 h with ice bath. The precipitate was washed with heptane (3 × 5 ml) to yield the title compound as an orange brown solid (20 mg, 41%). Red single crystals were grown in CHCl_3_/heptane mixture at 245 K for 1 day. ^1^H NMR (300 MHz), CDCl_3_): δ 5.71 (s, 6H, benzene), 2.79 (m, 2H, Cy), 2.06–1.54 (m, 14H, Cy), 1.27 (br s, 6H, Cy). ^13^C {^1^H} NMR (75 MHz, CDCl_3_): δ 89.8 (RuPh), 40.4 (d, *J_PC_* = 9.5 Hz, P*C*H), 27.2, 26.9, 26.7, 26.4, 26.3, 26.0, 26.0, 25.6 (*C*H_2_). ^31^P {^1^H} NMR (121 MHz, CDCl_3_): δ 156.3.

### S3. Refinement

H atoms were placed in idealized positions with C—H = 0.95–1.00 Å (CH), 0.99 Å (CH_2_) and refined using a riding model with *U*_iso_(H) fixed at 1.2*U*_eq_(C).

### Crystal data

**Table:**

[RuCl_2_(C_6_H_6_)(C_12_H_22_ClP)]·CHCl_3_	*F*(000) = 1216
*M**_r_* = 602.16	*D*_x_ = 1.705 Mg m^−^^3^
Monoclinic, *P*2_1_/*n*	Mo *K*α radiation, λ = 0.71073 Å
*a* = 7.9717 (1) Å	Cell parameters from 9877 reflections
*b* = 16.3020 (2) Å	θ = 2.3–27.9°
*c* = 18.0602 (3) Å	µ = 1.42 mm^−^^1^
β = 91.244 (1)°	*T* = 150 K
*V* = 2346.45 (6) Å^3^	Prism, orange
*Z* = 4	0.36 × 0.22 × 0.11 mm

### Data collection

**Table:**

Bruker Kappa APEXII DUO diffractometer	5619 independent reflections
Radiation source: fine-focus sealed tube	4963 reflections with *I* > 2σ(*I*)
Curved graphite monochromator	*R*_int_ = 0.031
Detector resolution: 8.3333 pixels mm^-1^	θ_max_ = 27.9°, θ_min_ = 1.7°
ω scans	*h* = −10→10
Absorption correction: multi-scan (*SADABS*; Bruker, 2008)	*k* = −21→21
*T*_min_ = 0.630, *T*_max_ = 0.859	*l* = −23→23
37052 measured reflections	

### Refinement

**Table:**

Refinement on *F*^2^	Primary atom site location: structure-invariant direct methods
Least-squares matrix: full	Secondary atom site location: difference Fourier map
*R*[*F*^2^ > 2σ(*F*^2^)] = 0.019	Hydrogen site location: inferred from neighbouring sites
*wR*(*F*^2^) = 0.048	H-atom parameters constrained
*S* = 1.05	*w* = 1/[σ^2^(*F*_o_^2^) + (0.0187*P*)^2^ + 1.4791*P*] where *P* = (*F*_o_^2^ + 2*F*_c_^2^)/3
5619 reflections	(Δ/σ)_max_ = 0.001
244 parameters	Δρ_max_ = 0.41 e Å^−^^3^
0 restraints	Δρ_min_ = −0.37 e Å^−^^3^

### Special details

**Table:**

Geometry. All e.s.d.'s (except the e.s.d. in the dihedral angle between two l.s. planes) are estimated using the full covariance matrix. The cell e.s.d.'s are taken into account individually in the estimation of e.s.d.'s in distances, angles and torsion angles; correlations between e.s.d.'s in cell parameters are only used when they are defined by crystal symmetry. An approximate (isotropic) treatment of cell e.s.d.'s is used for estimating e.s.d.'s involving l.s. planes.
Refinement. Refinement of *F*^2^ against ALL reflections. The weighted *R*-factor *wR* and goodness of fit *S* are based on *F*^2^, conventional *R*-factors *R* are based on *F*, with *F* set to zero for negative *F*^2^. The threshold expression of *F*^2^ > σ(*F*^2^) is used only for calculating *R*-factors(gt) *etc*. and is not relevant to the choice of reflections for refinement. *R*-factors based on *F*^2^ are statistically about twice as large as those based on *F*, and *R*- factors based on ALL data will be even larger.

### Fractional atomic coordinates and isotropic or equivalent isotropic displacement parameters (Å^2^)

**Table:**

	*x*	*y*	*z*	*U*_iso_*/*U*_eq_	
C1	−0.2498 (2)	0.37279 (11)	0.04879 (10)	0.0223 (4)	
H1	−0.3279	0.3746	0.0083	0.027*	
C2	−0.2406 (2)	0.30292 (11)	0.09373 (10)	0.0227 (4)	
H2	−0.3181	0.2592	0.0863	0.027*	
C3	−0.1149 (2)	0.29763 (11)	0.15045 (10)	0.0230 (4)	
H3	−0.1069	0.2501	0.1808	0.028*	
C4	−0.0024 (2)	0.36315 (11)	0.16134 (10)	0.0231 (4)	
H4	0.0864	0.3585	0.1970	0.028*	
C5	−0.0212 (2)	0.43653 (11)	0.11906 (10)	0.0235 (4)	
H5	0.0498	0.4822	0.1291	0.028*	
C6	−0.1430 (2)	0.44123 (11)	0.06332 (10)	0.0227 (4)	
H6	−0.1552	0.4900	0.0349	0.027*	
C7	0.2490 (2)	0.14568 (10)	0.07869 (9)	0.0160 (3)	
H7	0.3484	0.1741	0.0577	0.019*	
C8	0.2460 (2)	0.16860 (11)	0.16112 (9)	0.0211 (3)	
H8A	0.2381	0.2290	0.1661	0.025*	
H8B	0.1456	0.1442	0.1837	0.025*	
C9	0.4035 (2)	0.13820 (11)	0.20233 (10)	0.0238 (4)	
H9A	0.5032	0.1664	0.1828	0.029*	
H9B	0.3958	0.1518	0.2556	0.029*	
C10	0.4238 (2)	0.04591 (11)	0.19335 (11)	0.0276 (4)	
H10A	0.5280	0.0278	0.2193	0.033*	
H10B	0.3280	0.0174	0.2160	0.033*	
C11	0.4316 (2)	0.02332 (11)	0.11175 (11)	0.0260 (4)	
H11A	0.4399	−0.0370	0.1070	0.031*	
H11B	0.5337	0.0476	0.0905	0.031*	
C12	0.2773 (2)	0.05349 (10)	0.06779 (10)	0.0205 (3)	
H12A	0.1771	0.0231	0.0840	0.025*	
H12B	0.2924	0.0421	0.0145	0.025*	
C13	0.0709 (2)	0.13975 (10)	−0.06377 (9)	0.0161 (3)	
H13	0.0799	0.0791	−0.0565	0.019*	
C14	−0.0879 (2)	0.15535 (10)	−0.11130 (10)	0.0200 (3)	
H14A	−0.1863	0.1318	−0.0865	0.024*	
H14B	−0.1060	0.2152	−0.1166	0.024*	
C15	−0.0711 (2)	0.11651 (12)	−0.18795 (10)	0.0270 (4)	
H15A	−0.0682	0.0561	−0.1828	0.032*	
H15B	−0.1705	0.1310	−0.2190	0.032*	
C16	0.0864 (3)	0.14489 (13)	−0.22631 (10)	0.0305 (4)	
H16A	0.0799	0.2047	−0.2356	0.037*	
H16B	0.0953	0.1167	−0.2746	0.037*	
C17	0.2405 (2)	0.12592 (12)	−0.17829 (10)	0.0246 (4)	
H17A	0.3422	0.1453	−0.2035	0.030*	
H17B	0.2503	0.0658	−0.1715	0.030*	
C18	0.2299 (2)	0.16729 (10)	−0.10279 (9)	0.0193 (3)	
H18A	0.2282	0.2276	−0.1092	0.023*	
H18B	0.3298	0.1528	−0.0720	0.023*	
C19	0.4349 (2)	0.38768 (11)	0.85956 (10)	0.0222 (4)	
H19	0.3475	0.3760	0.8970	0.027*	
Cl1	0.01613 (5)	0.35589 (2)	−0.08323 (2)	0.01901 (8)	
Cl2	0.30706 (5)	0.34441 (2)	0.04619 (2)	0.02103 (9)	
Cl3	−0.12314 (5)	0.11221 (2)	0.07235 (2)	0.02052 (8)	
Cl4	0.54297 (6)	0.47752 (3)	0.88609 (3)	0.03089 (10)	
Cl5	0.57325 (6)	0.30361 (3)	0.85639 (3)	0.03164 (11)	
Cl6	0.33498 (7)	0.40171 (4)	0.77265 (3)	0.03799 (12)	
P1	0.06247 (5)	0.18613 (2)	0.02855 (2)	0.01377 (8)	
Ru1	0.007356 (16)	0.324634 (7)	0.046687 (7)	0.01356 (4)	

### Atomic displacement parameters (Å^2^)

**Table:**

	*U*^11^	*U*^22^	*U*^33^	*U*^12^	*U*^13^	*U*^23^
C1	0.0154 (8)	0.0266 (9)	0.0251 (9)	0.0050 (7)	0.0032 (7)	−0.0038 (7)
C2	0.0166 (8)	0.0240 (8)	0.0279 (9)	−0.0015 (7)	0.0095 (7)	−0.0046 (7)
C3	0.0270 (9)	0.0234 (8)	0.0190 (8)	0.0043 (7)	0.0099 (7)	0.0008 (7)
C4	0.0251 (9)	0.0275 (9)	0.0170 (8)	0.0056 (7)	0.0010 (7)	−0.0062 (7)
C5	0.0250 (9)	0.0204 (8)	0.0253 (9)	0.0017 (7)	0.0054 (7)	−0.0085 (7)
C6	0.0232 (9)	0.0191 (8)	0.0260 (9)	0.0067 (7)	0.0067 (7)	−0.0004 (7)
C7	0.0141 (7)	0.0165 (7)	0.0173 (8)	0.0005 (6)	−0.0004 (6)	0.0005 (6)
C8	0.0222 (8)	0.0236 (8)	0.0176 (8)	0.0027 (7)	−0.0016 (7)	−0.0017 (7)
C9	0.0224 (9)	0.0273 (9)	0.0213 (9)	−0.0005 (7)	−0.0066 (7)	0.0002 (7)
C10	0.0218 (9)	0.0260 (9)	0.0344 (10)	−0.0004 (7)	−0.0097 (8)	0.0074 (8)
C11	0.0202 (9)	0.0197 (8)	0.0378 (10)	0.0045 (7)	−0.0052 (8)	−0.0008 (8)
C12	0.0206 (8)	0.0159 (8)	0.0248 (9)	0.0015 (6)	−0.0029 (7)	−0.0004 (7)
C13	0.0174 (8)	0.0145 (7)	0.0165 (8)	−0.0002 (6)	0.0008 (6)	−0.0024 (6)
C14	0.0196 (8)	0.0189 (8)	0.0213 (8)	−0.0024 (6)	−0.0035 (7)	−0.0011 (6)
C15	0.0309 (10)	0.0306 (10)	0.0192 (9)	−0.0067 (8)	−0.0079 (7)	0.0000 (7)
C16	0.0403 (11)	0.0340 (10)	0.0171 (9)	−0.0065 (9)	−0.0011 (8)	0.0025 (8)
C17	0.0279 (9)	0.0283 (9)	0.0178 (8)	0.0010 (8)	0.0057 (7)	−0.0024 (7)
C18	0.0196 (8)	0.0216 (8)	0.0168 (8)	0.0000 (6)	0.0015 (6)	−0.0015 (6)
C19	0.0174 (8)	0.0282 (9)	0.0210 (8)	0.0003 (7)	0.0000 (7)	0.0001 (7)
Cl1	0.02313 (19)	0.01688 (18)	0.01704 (19)	−0.00012 (15)	0.00118 (15)	0.00162 (14)
Cl2	0.01470 (18)	0.02085 (19)	0.0275 (2)	−0.00415 (15)	0.00011 (16)	−0.00070 (16)
Cl3	0.01850 (18)	0.01891 (19)	0.0243 (2)	−0.00493 (15)	0.00428 (15)	0.00082 (15)
Cl4	0.0287 (2)	0.0250 (2)	0.0388 (3)	−0.00293 (18)	−0.0043 (2)	−0.00117 (19)
Cl5	0.0251 (2)	0.0273 (2)	0.0425 (3)	0.00382 (18)	0.0000 (2)	−0.0002 (2)
Cl6	0.0334 (3)	0.0535 (3)	0.0266 (2)	0.0079 (2)	−0.0096 (2)	−0.0028 (2)
P1	0.01298 (18)	0.01378 (18)	0.01459 (19)	−0.00112 (15)	0.00104 (15)	−0.00075 (15)
Ru1	0.01285 (6)	0.01339 (6)	0.01447 (6)	−0.00015 (5)	0.00123 (5)	−0.00095 (5)

### Geometric parameters (Å, º)

**Table:**

C1—C2	1.400 (3)	C11—C12	1.530 (2)
C1—C6	1.425 (2)	C11—H11A	0.9900
C1—Ru1	2.1966 (17)	C11—H11B	0.9900
C1—H1	0.9500	C12—H12A	0.9900
C2—C3	1.420 (3)	C12—H12B	0.9900
C2—Ru1	2.1970 (17)	C13—C18	1.530 (2)
C2—H2	0.9500	C13—C14	1.535 (2)
C3—C4	1.406 (3)	C13—P1	1.8334 (16)
C3—Ru1	2.1759 (17)	C13—H13	1.0000
C3—H3	0.9500	C14—C15	1.531 (2)
C4—C5	1.425 (3)	C14—H14A	0.9900
C4—Ru1	2.1668 (17)	C14—H14B	0.9900
C4—H4	0.9500	C15—C16	1.519 (3)
C5—C6	1.386 (3)	C15—H15A	0.9900
C5—Ru1	2.2585 (17)	C15—H15B	0.9900
C5—H5	0.9500	C16—C17	1.520 (3)
C6—Ru1	2.2705 (17)	C16—H16A	0.9900
C6—H6	0.9500	C16—H16B	0.9900
C7—C12	1.533 (2)	C17—C18	1.525 (2)
C7—C8	1.536 (2)	C17—H17A	0.9900
C7—P1	1.8457 (16)	C17—H17B	0.9900
C7—H7	1.0000	C18—H18A	0.9900
C8—C9	1.528 (2)	C18—H18B	0.9900
C8—H8A	0.9900	C19—Cl6	1.7596 (18)
C8—H8B	0.9900	C19—Cl4	1.7602 (18)
C9—C10	1.522 (3)	C19—Cl5	1.7609 (18)
C9—H9A	0.9900	C19—H19	1.0000
C9—H9B	0.9900	Cl1—Ru1	2.4036 (4)
C10—C11	1.522 (3)	Cl2—Ru1	2.4110 (4)
C10—H10A	0.9900	Cl3—P1	2.0779 (6)
C10—H10B	0.9900	P1—Ru1	2.3247 (4)
			
C2—C1—C6	120.45 (17)	C15—C14—C13	110.48 (14)
C2—C1—Ru1	71.44 (10)	C15—C14—H14A	109.6
C6—C1—Ru1	74.25 (10)	C13—C14—H14A	109.6
C2—C1—H1	119.8	C15—C14—H14B	109.6
C6—C1—H1	119.8	C13—C14—H14B	109.6
Ru1—C1—H1	126.4	H14A—C14—H14B	108.1
C1—C2—C3	119.69 (16)	C16—C15—C14	112.07 (15)
C1—C2—Ru1	71.41 (10)	C16—C15—H15A	109.2
C3—C2—Ru1	70.25 (10)	C14—C15—H15A	109.2
C1—C2—H2	120.2	C16—C15—H15B	109.2
C3—C2—H2	120.2	C14—C15—H15B	109.2
Ru1—C2—H2	130.8	H15A—C15—H15B	107.9
C4—C3—C2	119.52 (17)	C15—C16—C17	110.09 (15)
C4—C3—Ru1	70.76 (10)	C15—C16—H16A	109.6
C2—C3—Ru1	71.86 (10)	C17—C16—H16A	109.6
C4—C3—H3	120.2	C15—C16—H16B	109.6
C2—C3—H3	120.2	C17—C16—H16B	109.6
Ru1—C3—H3	129.5	H16A—C16—H16B	108.2
C3—C4—C5	120.24 (17)	C16—C17—C18	111.10 (15)
C3—C4—Ru1	71.47 (10)	C16—C17—H17A	109.4
C5—C4—Ru1	74.74 (10)	C18—C17—H17A	109.4
C3—C4—H4	119.9	C16—C17—H17B	109.4
C5—C4—H4	119.9	C18—C17—H17B	109.4
Ru1—C4—H4	125.7	H17A—C17—H17B	108.0
C6—C5—C4	119.95 (17)	C17—C18—C13	110.17 (14)
C6—C5—Ru1	72.66 (10)	C17—C18—H18A	109.6
C4—C5—Ru1	67.75 (9)	C13—C18—H18A	109.6
C6—C5—H5	120.0	C17—C18—H18B	109.6
C4—C5—H5	120.0	C13—C18—H18B	109.6
Ru1—C5—H5	132.5	H18A—C18—H18B	108.1
C5—C6—C1	119.83 (17)	Cl6—C19—Cl4	110.13 (10)
C5—C6—Ru1	71.71 (10)	Cl6—C19—Cl5	110.11 (10)
C1—C6—Ru1	68.61 (9)	Cl4—C19—Cl5	110.69 (9)
C5—C6—H6	120.1	Cl6—C19—H19	108.6
C1—C6—H6	120.1	Cl4—C19—H19	108.6
Ru1—C6—H6	132.6	Cl5—C19—H19	108.6
C12—C7—C8	111.62 (14)	C13—P1—C7	104.70 (7)
C12—C7—P1	114.00 (11)	C13—P1—Cl3	98.49 (5)
C8—C7—P1	111.07 (11)	C7—P1—Cl3	100.29 (6)
C12—C7—H7	106.5	C13—P1—Ru1	122.65 (5)
C8—C7—H7	106.5	C7—P1—Ru1	115.50 (5)
P1—C7—H7	106.5	Cl3—P1—Ru1	111.79 (2)
C9—C8—C7	111.31 (14)	C4—Ru1—C3	37.77 (7)
C9—C8—H8A	109.4	C4—Ru1—C1	80.04 (7)
C7—C8—H8A	109.4	C3—Ru1—C1	67.77 (7)
C9—C8—H8B	109.4	C4—Ru1—C2	68.03 (7)
C7—C8—H8B	109.4	C3—Ru1—C2	37.89 (7)
H8A—C8—H8B	108.0	C1—Ru1—C2	37.15 (7)
C10—C9—C8	110.91 (15)	C4—Ru1—C5	37.51 (7)
C10—C9—H9A	109.5	C3—Ru1—C5	67.19 (7)
C8—C9—H9A	109.5	C1—Ru1—C5	66.15 (7)
C10—C9—H9B	109.5	C2—Ru1—C5	78.67 (7)
C8—C9—H9B	109.5	C4—Ru1—C6	66.48 (7)
H9A—C9—H9B	108.0	C3—Ru1—C6	78.92 (7)
C11—C10—C9	110.42 (15)	C1—Ru1—C6	37.15 (6)
C11—C10—H10A	109.6	C2—Ru1—C6	66.53 (7)
C9—C10—H10A	109.6	C5—Ru1—C6	35.63 (7)
C11—C10—H10B	109.6	C4—Ru1—P1	115.27 (5)
C9—C10—H10B	109.6	C3—Ru1—P1	90.82 (5)
H10A—C10—H10B	108.1	C1—Ru1—P1	121.93 (5)
C10—C11—C12	112.06 (15)	C2—Ru1—P1	94.15 (5)
C10—C11—H11A	109.2	C5—Ru1—P1	152.59 (5)
C12—C11—H11A	109.2	C6—Ru1—P1	159.01 (5)
C10—C11—H11B	109.2	C4—Ru1—Cl1	150.91 (5)
C12—C11—H11B	109.2	C3—Ru1—Cl1	155.05 (5)
H11A—C11—H11B	107.9	C1—Ru1—Cl1	89.32 (5)
C11—C12—C7	111.57 (14)	C2—Ru1—Cl1	117.20 (5)
C11—C12—H12A	109.3	C5—Ru1—Cl1	113.53 (5)
C7—C12—H12A	109.3	C6—Ru1—Cl1	88.76 (5)
C11—C12—H12B	109.3	P1—Ru1—Cl1	93.366 (15)
C7—C12—H12B	109.3	C4—Ru1—Cl2	91.20 (5)
H12A—C12—H12B	108.0	C3—Ru1—Cl2	119.52 (5)
C18—C13—C14	112.01 (14)	C1—Ru1—Cl2	151.36 (5)
C18—C13—P1	110.18 (11)	C2—Ru1—Cl2	157.35 (5)
C14—C13—P1	113.20 (11)	C5—Ru1—Cl2	90.37 (5)
C18—C13—H13	107.0	C6—Ru1—Cl2	114.49 (5)
C14—C13—H13	107.0	P1—Ru1—Cl2	86.505 (14)
P1—C13—H13	107.0	Cl1—Ru1—Cl2	85.306 (15)

### Hydrogen-bond geometry (Å, º)

**Table:**

*D*—H···*A*	*D*—H	H···*A*	*D*···*A*	*D*—H···*A*
C14—H14*B*···Cl1	0.99	2.56	3.4080 (17)	144
C18—H18*A*···Cl1	0.99	2.74	3.5366 (17)	138
C19—H19···Cl1^i^	1.00	2.69	3.5539 (17)	144
C19—H19···Cl2^i^	1.00	2.77	3.6119 (18)	142

Symmetry code: (i) *x*, *y*, *z*+1.

## References

[bb1] Bruker (2008). *SADABS* Bruker AXS Inc., Madison, Wisconsin, USA.

[bb2] Bruker (2009). *SAINT* Bruker AXS Inc., Madison, Wisconsin, USA.

[bb3] Bruker (2011). *APEX2* Bruker AXS Inc., Madison, Wisconsin, USA.

[bb5] Jantscher, F., Kirchner, K. & Mereiter, K. (2009). *Acta Cryst.* E**65**, m941. 10.1107/S1600536809027676 PMC2977503 21583392

[bb6] Sheldrick, G. M. (2008). *Acta Cryst.* A**64**, 112–122. 10.1107/S0108767307043930 18156677

[bb7] Torres-Lubián, R., Rosales-Hoz, M. J., Arif, A. M., Ernst, R. D. & Paz-Sandoval, M. A. (1999). *J. Organomet. Chem.* **585**, 68–82.

